# MRI Observation After Intralabyrinthine and Vestibular Schwannoma Resection and Cochlear Implantation

**DOI:** 10.3389/fneur.2020.00759

**Published:** 2020-08-12

**Authors:** Holger Sudhoff, Hans B. Gehl, Lars U. Scholtz, Ingo Todt

**Affiliations:** ^1^Department of Otolaryngology, Head and Neck Surgery, Klinikum Bielefeld, Bielefeld University, Bielefeld, Germany; ^2^Department of Radiology, Klinikum Bielefeld, Bielefeld, Germany

**Keywords:** MRI, cochlear implant, vestibular schwannoma, intralabyrinthine schwannoma, tumor follow up

## Abstract

**Objective:** MRI observation is part of the regular follow-up after vestibular schwannoma (VS) or intralabyrinthine schwannoma (ILS) resection. Because cochlear implantation (CI) after resection is part of the audiological rehabilitation process, the magnet resonance imaging (MRI) behavior of CI systems needs to be considered. In light of recent developments in MRI artifact positioning and pain prevention, this study evaluates reproducible MRI observations after tumor resection and CI surgery as part of follow-up.

**Methods:** In a retrospective study, we evaluated 9 patients with a T1 KM, T2 sequence MRI observation, and cone beam computed tomography (CBCT) after ILS/VS resection and CI. In all but one case, a CI with a diametrically bipolar magnet and a receiver positioned 8–9 cm behind the external auditory canal was performed.

**Results:** In all but one case, MRI observation allowed for a pain-free visual assessment of the intralabyrinthine and internal auditory canal (IAC) regions. In one case, a painful dislodgement of the receiver magnet occurred.

**Conclusion:** MRI follow-up after ILS and VS resection and CI is reproducibly possible. Implant choice and positioning should be considered before implantation to allow for a pain-free visual assessment afterward. This finding allows for the first time a widening of the indication into this patient group.

## Introduction

Cochlear implantation (CI) is the method of choice when treating cases of severe to profound hearing loss that do not show sufficient improvement in speech understanding with the use of hearing aids. Even in cases of unilateral hearing loss or deafness, CI has been shown to restore hearing and hearing localization (1, 2).

Unilateral hearing loss can be caused by a vestibular schwannoma (VS) or intralabyrinthine schwannoma (ILS). Therefore, CI has received attention as a possible treatment option for hearing rehabilitation.

CI has been shown to be a successful treatment option in cases of ILS (3–6) and VS (7–9). While CI is regularly performed as a single-stage procedure in cases of ILS (5, 6), CI in VS can be performed as a single-stage procedure based on the visual assessment of cochlear nerve integrity (8) or based on unreliable ABR measurement. It is otherwise performed as a two-stage procedure that includes both a pre-surgery MRI to minimize the risk of a residual tumor and promontorial testing after a year to semi-objectively test nerve function (9).

Regular tumor follow-up for cases of VS and ILS resection in combination with CI has been discussed (10), but the problem has so far not been solved.

MRI observations have long been contraindicated after CI (11). With the introduction of headbands, a compromise was found between the need for an MRI scan and the risk of pain and magnet dislocation. Depending on the internal magnet used in the implant, MRI scans can be associated with complications such as pain, demagnetization, artifacts, and magnet dislodgements (12–16). Additionally, scans at 3 Tesla (3 T) cannot be performed without the removal of the magnet (Neuro 2, Oticon, Vallauris, France). This limits the usability of MRI scans. New implants containing diametrically bipolar magnets (Synchrony, MED-EL, Austria, Innsbruck) allow for pain-free MRI scans at 3 T, even without a headband (17).

Recent studies demonstrated that an assessment of the tumor region is possible in cases of NF II (18). Todt et al. showed that the axial assessment of the internal auditory canal (IAC) and the cochlea is dependent upon the specific positioning of the implant magnet and MRI sequence even after implantation (19). Carlson showed the possibility of a coronal assessment (20). Further important factors are artifact-reducing sequences (e.g., MARS) (21) and the position of the head in the MRI scanner (22).

These observations allow for a visual assessment of the cochlea and IAC after initial electrode insertion.

The aim of this study was to evaluate the follow-up assessment of the ipsilateral cochlea and IAC after ILS and VS resection and CI surgery.

## Materials and Methods

In this study, 8 out of 9 patients underwent a 3-T MRI scan in a tertiary referral center. In one case, a 1.5-T MRI was performed. Between 5/2017 and 8/2019, 7 patients were implanted with a MED-EL SYNCHRONY implant (MED-EL, Innsbruck, Austria) with a diametrically magnetized internal magnet (6 ILS cases and 1 VS case). In one ILS case, a Cochlear 512 Profile (Cochlear, Sydney, Australia) and in one VS case, an Advanced Bionics 3D (Advanced Bionics, Stäfa, Swiss) was implanted. In most of the ILS cases, a single-stage procedure was performed (Table 1). In the two VS cases, a two-stage procedure was performed, and the indication for CI was based on positive promontorial testing. One VS cases had previously undergone surgery using a translabyrinthine approach. The other cases involved a retrosigmoidal approach. In all cases, the implant magnet was intraoperatively determined and positioned 7–9 cm behind the external auditory canal (19).

**Table 1:** Description of individual data.

**Patient**	**Tumor**	**Tumor size and position**	**Implant**	**Stage**	**Period between resection and CI**	**Period between** **resection and last MRI**	**Recurrence of Tumor or residuum**	**Visual assessment of IAC**	**Visual assessment of cochlea**	**Tumor approach, Surgical procedure**
1	Intralabyrinthine schwannoma, vestibulum	1 x 1,5 mm and partial upper and lateral semicircilar canal	Synchrony	2 stage	9 y	9 y	No	++	++	Translabyrinthine, 1)
2	Intralabyrinthine schwannoma, cochlea	Scala tympani, basal turn	512 > pain and dislodgement	1 stage		1 d		++	++	Posterior tympanotomy, 4)
3	Intralabyrinthine schwannoma, vestibulum	3 x 3,5 mm	Synchrony	1 stage	3 y	3 y	No	++	++	Translabyrinthine, 1)
4	Intralabyrinthine schwannoma, vestibulum	3 x 3,5 mm	Synchrony	2 stage	8 y	8 y	No	++	++	Translabyrinthine, 1)
5	Intralabyrinthine schwannoma, cochlea	Full cochlea	Synchrony	1 stage		1 d		++	++	Transotic, 2)
6	Intralabyrinthine schwannoma, cochlea	Scala tympani basal and first turn	Synchrony	1 stage		1 d		++	++	Posterior tympanotomy, 3)
7	Intralabyrinthine schwannoma, vestibulum	4 x 4mm	Synchrony	1 stage		1 d		++	++	Translabyrinthine, 1)
8	Vestibular schwannoma,	Intrameatal 9 x 4 mm	Synchrony	2 stage	2 y	2 y	No	++	++	Translabyrinthine
9	Vestibular schwannoma	Intrameatal 10 x 5 mm	Advanced Bionics 3D	2 stage	3 y	2 y	No	++	++	Retrosigmoidal

*Data show tumor location and specific size; implant brand and type; staged surgical access; time period between tumor resection and CI surgery; time period between resection and last MRI; recurrence or residual tumor; visual assessment is scaled from no visualization: O; cloudy visualization: +; no limitation in terms of visualization: ++; the surgical access to remove the tumor is described. Performed schwannoma removal techniques were 1) labyrinthectomy; 2) cochleostomy in combination with a first turn access to the cochlea; 3) drill-out or subtotal cochlectomy in combination with a subtotal petrosectomy and blind sac closure; 4) enlarged cochleostomy*.

On the first postoperative day, examinations were performed without a headband on the MED-EL SYNCHRONY and Advanced Bionics 3D cases using 3-T and 1.5-T MRI units (Achieva, Philips Medical Systems, Best, NL). A headband was used with the Cochlear 512 case.

Sequence: T1/ T1 Gad, slice thickness 2 mm, resolution: 0.6 × 0.8 mm, FOV 150 × 150, TE 70, TR 3000, TSE 8, Sham Spir.

A specific artifact reduction sequence was not used.

Additionally, a CBCT (NewTom VGI, Verona, Italy) or multislice computed tomography (MSCT) 80 was performed for the estimation of the electrode position in all cases. CBCT parameters were as follows.

FOV 15 × 15 cm, 10.48 mAS-20.52 mAS, KV 110, 360° followed by 2D and 3D reconstructions at an external workstation (NNT, main station).

The MSCT Toshiba Aquilion 80 protocol was as follows: slice thickness 0.5 mm, KV 120, MA 200, rot. time 0.75.

In all cases, a T2w and a t1 /T1 Gad. sequence was performed in axial and coronal plain. MRI image evaluation was performed independently by a neuroradiologist and a surgeon. We observed no significant differences in terms of MRI artifact size between T2w and T1/T1 Gad sequences. Visual assessment of IAC and Cochlea was descriptively scaled from the following: no visualization: O; cloudy visualization: +; no limitation in terms of visualization: ++. Since there was no variance, an applicable *t*-test could not be performed. The information about the technique of surgical tumor removal is given in Table 1.

## Results

All MED-EL SYNCHRONY and the single AB 3D patient underwent MRI scanning without pain or discomfort. In the case of Cochlear 512, a magnet dislocation occurred. This was corrected by a second surgery.

In all cases, the cochlea and the IAC were visually accessible. The visual assessment of the structures of interest was not limited by the implant artifact or artifact torsion. In all two-stage surgeries, no recurrence or residual schwannoma could be observed.

**Figure 1:**
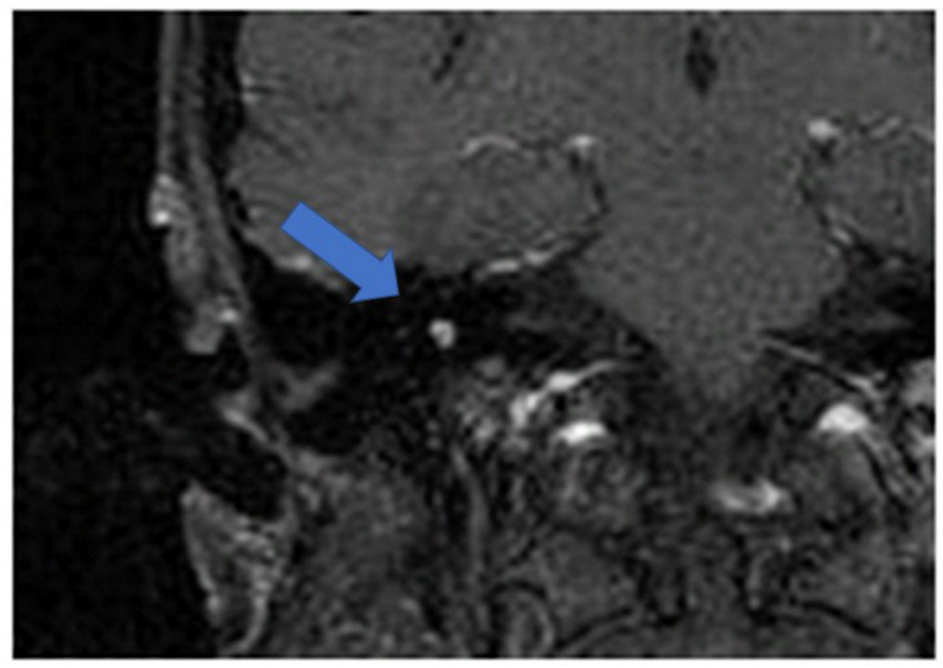
An exemplary ILS tumor (T1, KM), coronal. Arrow indicates tumor.

**Figure 2:**
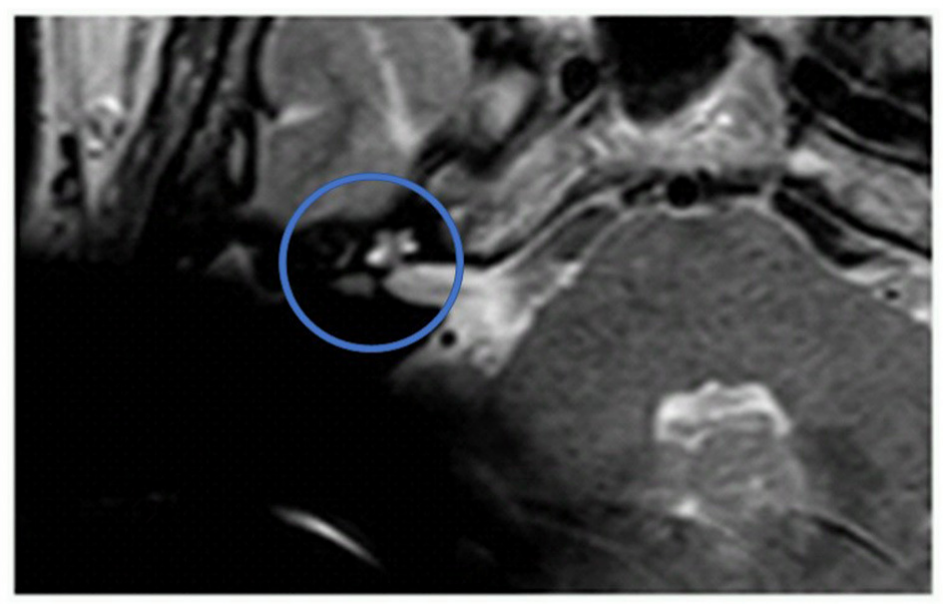
The ILS region with a T2 TSE sequence after CI, axial. Visual assessment of cochlea and vestibulum. Circle indicates region of interest.

**Figure 3:**
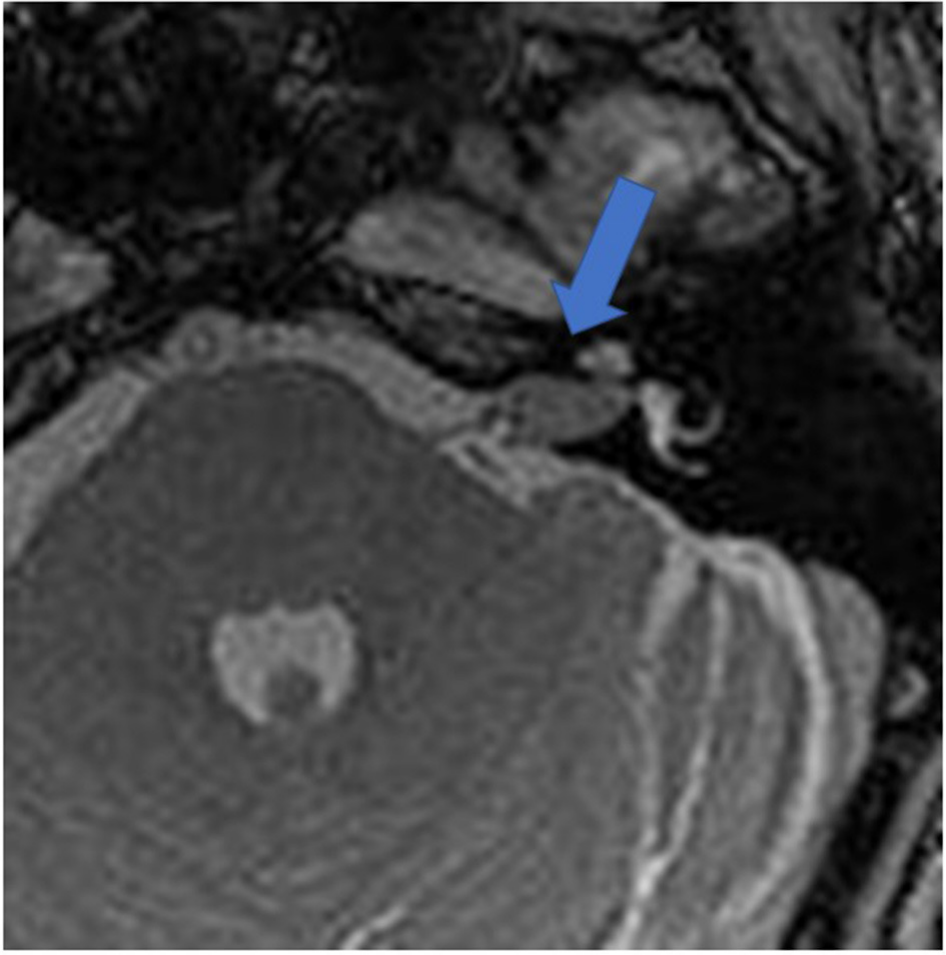
The exemplary VS before resection (T2 TSE). Arrow indicates tumor.

**Figure 4:**
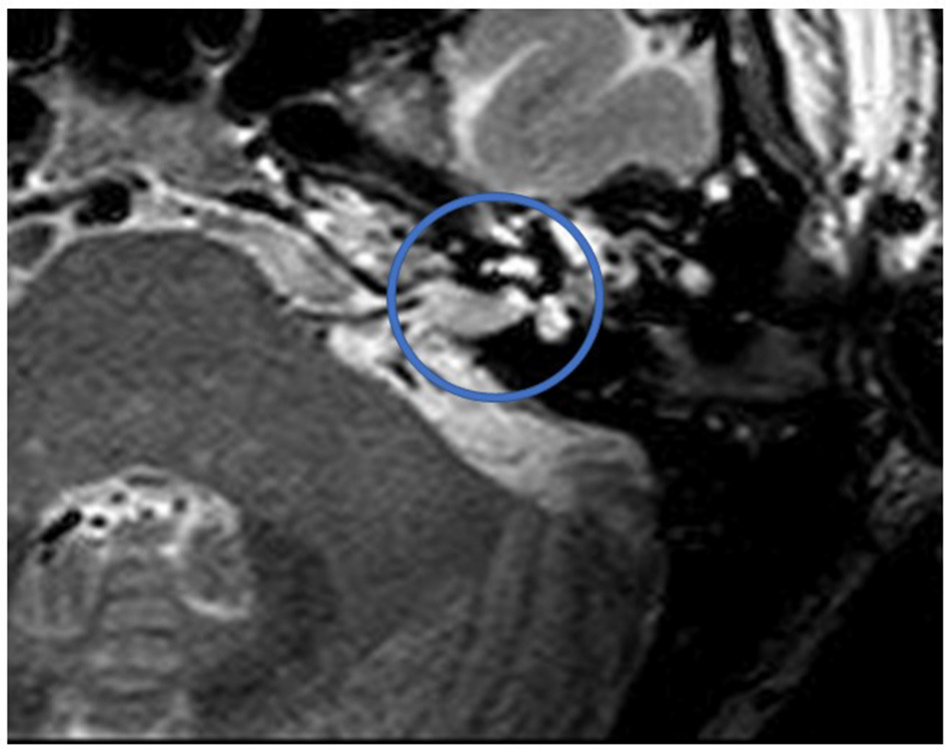
T2 TSE after CI, axial. Visual assessment of IAC. Circle indicates region of interest.

**Figure 5:**
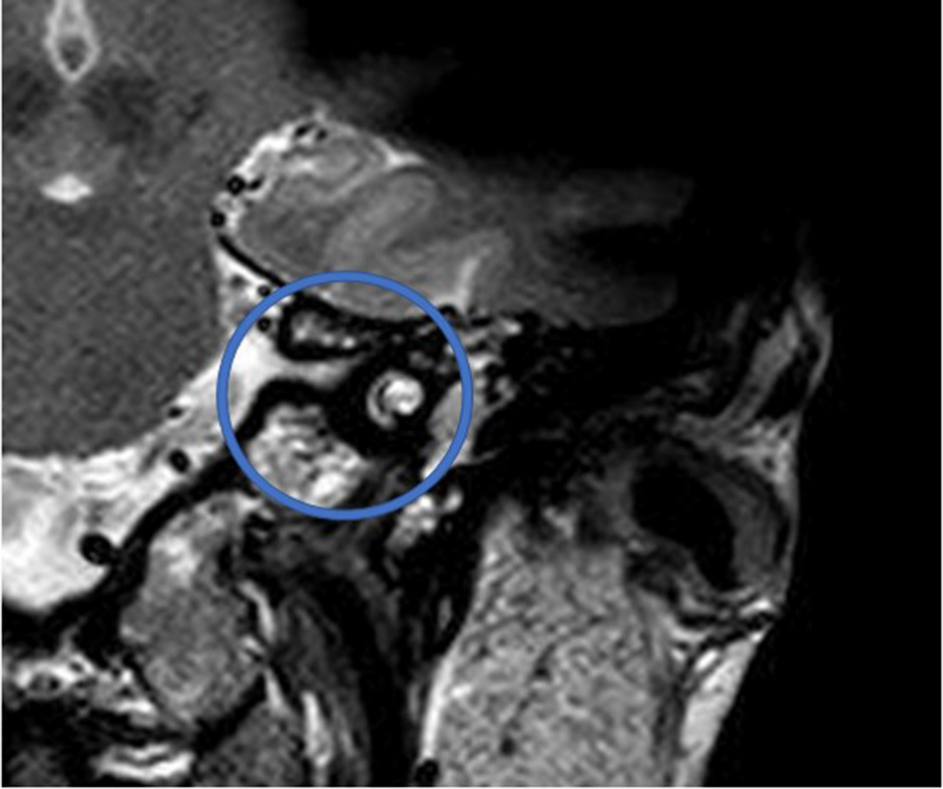
T2 TSE after CI, coronal. Visual assessment of cochlea. Circle indicates region of interest.

Figure 1 shows an exemplary ILS tumor (T1, KM) in the coronal plain. Figure 2 shows the ILS region with a T2 TSE sequence after CI, in the axial plain. Figure 3 shows the exemplary VS before resection (T1 KM) and Figure 4 the T2 TSE after CI in the axial and coronal plains (Figure 5). Individual data are presented in Table 1.

## Discussion

With the broadening of indication for CI for cases of unilateral deafness (1, 2), VS and ILS have become subjects of interest. ILS resection allows for a single-stage procedure with stable CI results (5, 6). The rate of residual ILS tumors after initial resection has so far not been published, but given that the procedure is conducted in a mostly pure bony anatomic environment, the rate can be assumed to be lower than in VS cases. However, an MRI-based tumor follow-up should be part of the CI rehabilitation concept.

A series on NF II CI was published in 2014 (18), demonstrating that imaging of the IAC is possible. Different approaches in cases of VS in combination with tumor removal have been performed in a single-stage procedure with CI (8). The integrity of the acoustic nerve was mostly evaluated by visual control or ABR and was therefore, to some degree, uncertain. Two approaches are used to solve this problem. One option is the intraoperative insertion of a cochlear probe in the case of the translabyrinthine approach and subsequent CI (23). The other is a two-stage procedure (9). One year after implantation, a promontorial test is performed to exclude the risk of nonfunctional acoustic nerve with a non-functional CI. A follow-up MRI scan minimizes the risk of residual VS before CI magnet artifacts further diminish visual evaluation of the tumor region.

An MRI-based tumor follow-up 5 years after VS resection is part of the CNC guidelines for VS treatment (24).

Recent developments in CI magnets [bipolar diametrical magnets (Medel Synchrony), 3D magnets (Advanced Bionics 3D)], and surgical techniques (implant positioning) have had a significant impact on the relationship between CI and MRI, as this study shows.

**Figure 6:**
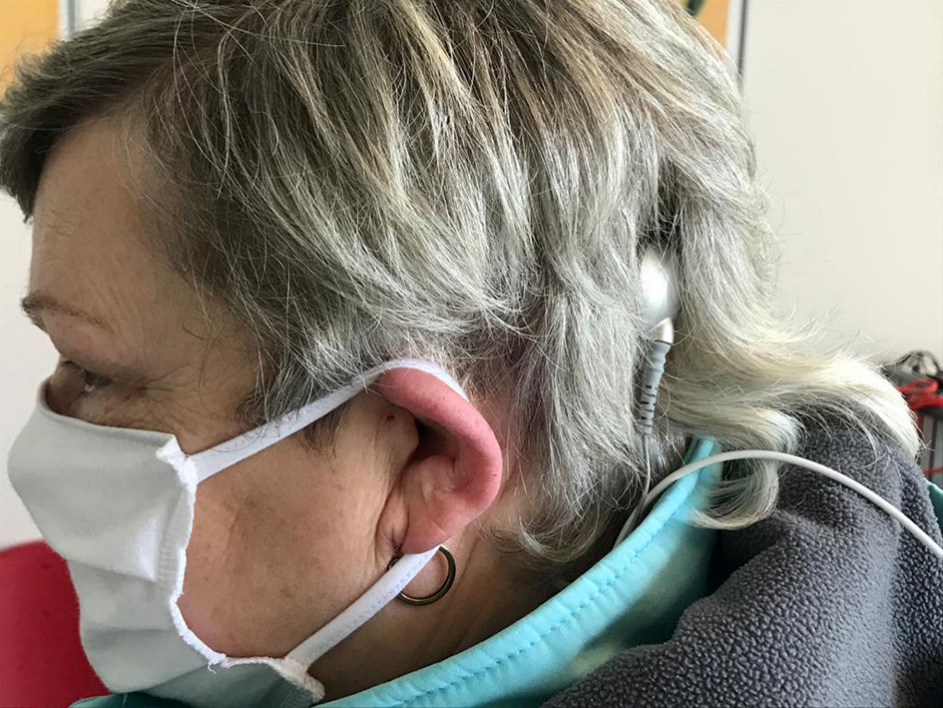
Exemplary recommended position of the implant with a nasion–external auditory canal–magnet angle of 140° and a distance of 9 cm.

In addition to the pain-free performance of MRI scanning even at 3 T, MRI scanning has become a valuable tool after CI. For the first time, specific implant positioning allows for a postsurgical visualization of the cochlea and IAC and, therefore, a tumor follow-up for cases of ILS/VS and CI. Besides the right choice of MRI sequence, the position of the implant 7–9 cm from the external auditory canal at an angle of 90 or 160° was shown to enable access to the cochlea and IAC (19) (Figure 6). SEMAC-VAT (Siemens), MAVRIC (General Electric), and O-MAR (Philips) are metal artifact reduction sequences (MARS) and should be further investigated to evidence their value for cochlear implant patients (21). The disadvantages of this sequence are their costs and a limitation in terms of combination with other sequences. Additionally, a limitation in resolution is discussed but needs further evaluation.

Additionally, a head position inside the scanner with the chin to the chest allows for a better visualization in the coronal plain of the structures of interest (22).

Imaging-based visualization of the cochlea after CI allows for the estimation of perimodiolar electrode position (25) and the localization of lateral wall electrodes in the basal turn (26) when performing this specific positioning. Postsurgical MRI scanning has been shown to identify brainstem infarction, which explains the new occurrence of vertigo after CI in some cases (27).

Positioning a bipolar diametrical implant enables visual control of the tumor region, as shown in this study. This can be performed without pain, as shown previously. Magnet dislocation occurred in the Cochlear 512 case, even with a headband, and surgical replacement of the magnet was necessary. This emphasizes the importance of magnet/implant choice. The consideration of the described requirements allows for the first time a reproduceable tumor follow-up. By this, a widening of the indication of cochlear implantation into intralabyrinthine and vestibular schwannoma hearing rehabilitation including a pain-free tumor follow-up is possible.

One limitation of this study is the small number of surgeries.

## Conclusion

MRI follow-up after ILS and VS resection and CI is reproducibly possible in our group. Implant choice and positioning should be considered to allow for a pain-free MRI scanning and visual assessment afterward. Based on our experience an MRI follow-up should be reproducibly possible even in larger groups.

## Data Availability Statement

The raw data supporting the conclusions of this article will be made available by the authors, without undue reservation.

## Ethics Statement

The studies involving human participants were reviewed and approved by the institutional review board of the Klinikum Bielefeld, Germany (IRB-klibi-HNO-2017/09). Patients provided their written informed consent for the use of their clinical records in this study.

## Disclosure

The authors received grants in the last 3 years from Advanced Bionics, MED-El, and Cochlear.

## Author Contributions

HS: head of bielefeld otola department and co-writing. HG: head of bielefeld radiology, discussion of radiological results, and co-writing. LS: consultant co-writing. IT: consultant, idea, surgeon, and writing. All authors contributed to the article and approved the submitted version.

## Conflict of Interest

The authors declare that the research was conducted in the absence of any commercial or financial relationships that could be construed as a potential conflict of interest.
